# Resistin Induces LIN28A-Mediated Let-7a Repression in Breast Cancer Cells Leading to IL-6 and STAT3 Upregulation

**DOI:** 10.3390/cancers13184498

**Published:** 2021-09-07

**Authors:** Sachin Kumar Deshmukh, Sanjeev Kumar Srivastava, Haseeb Zubair, Mohammad Aslam Khan, Ajay Pratap Singh, Seema Singh

**Affiliations:** 1 Department of Pathology, College of Medicine, University of South Alabama, Mobile, AL 36617, USA; skdeshmukh@southalabama.edu (S.K.D.); SSriva57@its.jnj.com (S.K.S.); Haseeb.Zubair@STJUDE.org (H.Z.); makhan@southalabama.edu (M.A.K.); asingh@southalabama.edu (A.P.S.); 2 Cancer Biology Program, Mitchell Cancer Institute, University of South Alabama, Mobile, AL 36604, USA; 3 Department of Biochemistry and Molecular Biology, College of Medicine, University of South Alabama, Mobile, AL 36688, USA

**Keywords:** breast cancer, resistin, Let-7a, LIN28A, STAT3, IL-6

## Abstract

**Simple Summary:**

Breast cancer is the second leading cause of cancer-related death in women in the United States and exhibits significant racial disparities in clinical outcomes. Earlier, we reported that the levels of resistin and IL-6 were significantly more elevated in the serum of African American women with breast cancer than in their Caucasian American counterparts. Here, we uncover its mechanistic significance by characterizing a novel resistin/LIN28A/Let-7a/IL-6/STAT3 signaling axis supporting the growth and stemness of breast cancer cells.

**Abstract:**

Downregulation of the Let-7 family of microRNAs (miRNAs) has been reported in several cancers, including breast malignancy; however, underlying mechanisms are not completely understood. Resistin is an important component of the tumor microenvironment, having a functional impact on the tumor cell phenotypes. Here, we examined the role of resistin in the regulation of Let-7 miRNAs and studied its downstream consequences. We found that resistin treatment led to the reduced expression of Let-7 family miRNAs in breast cancer (BC) cells, with the highest downregulation reported for Let-7a. Furthermore, resistin induced the expression of LIN28A, and its silencing abrogated resistin-mediated Let-7a suppression. Let-7a restoration or LIN28A silencing abolished the resistin-induced growth, clonogenicity, and sphere-forming ability of BC cells. Restoration of Let-7a also suppressed the resistin-induced expression of genes associated with growth, survival, and stemness. Pathway analysis suggested STAT3 as a putative central node associated with Let-7a-mediated gene regulation. In silico analysis identified STAT3 and its upstream modifier, IL-6, as putative Let-7a gene targets, which were later confirmed by 3′UTR-reporter assays. Together, our findings demonstrate a novel resistin/LIN28A/Let-7a/IL-6/STAT3 signaling axis supporting the growth and stemness of BC cells.

## 1. Introduction

Breast cancer (BC) is the most commonly diagnosed non-cutaneous malignancy and the foremost cause of cancer-related death in women in the United States [1]. The American Cancer Society estimates ~281,550 new diagnoses of BC this year in the United States, and nearly 43,600 women will die because of it [1]. Moreover, race-associated disparities in BC incidence and mortality remain a significant concern [2,3]. Therefore, it is extremely important to improve our understanding of breast tumor biology and delineate the molecular causes underlying disparate clinical outcomes among patients. Such information can help develop novel mechanism-based therapies and deliver them to the targeted patient population stratified based on the molecular features of the cancer.

MicroRNAs (miRNAs) are small (~22-nucleotides) noncoding RNAs that play crucial roles in gene regulation. miRNAs bind to the complementary sequences in the 3′ untranslated region (UTR) of the target mRNAs and cause either their degradation or inhibit translation [4,5]. Aberrant expression of miRNAs has been reported in almost all cancers and is associated with their pathobiology [6,7]. The Let-7 (lethal-7) family is among the first reported miRNAs and is evolutionarily conserved across various animal species [8,9,10]. Multiple mature Let-7 isoforms have been described in humans, regulated by multiple mechanisms, including the post-transcriptional regulation by two highly conserved RNA-binding proteins, LIN28A and LIN28B [11,12,13,14]. In addition, an aberrant expression of the Let-7 family of miRNAs has been reported in BC and shown to be involved in disease progression and therapy resistance [15,16].

Tumor microenvironment plays a significant role in cancer biology and clinical outcomes by having a profound impact on tumor cell behavior and its response to various therapies [17]. It is also emerging as a major player in racial health disparities [18,19,20]. Resistin is an inflammatory cytokine that has been associated with several pathological conditions and plays multi-faceted roles in cancer progression [21,22]. We previously reported racially disparate increased serum levels of resistin in BC patients and demonstrated its pathobiological significance [19,23]. Here, we investigated the effect of resistin treatment on the expression of Let-7 miRNAs in BC cells and their role in mediating its effects on BC cell phenotypes. Our findings reveal a novel resistin-induced, LIN28A-mediated mechanism for the regulation of Let-7a miRNA in BC cells and demonstrate its functional significance. We also demonstrate, for the first time, that resistin-induced Let-7a downregulation is associated with induced expression of STAT3 and IL6 in BC cells by directly targeting of their 3′ untranslated regions. Thus, our findings reveal a novel resistin-LIN28A-Let-7a-IL-6/STAT3 signaling axis implicated in BC pathobiology.

## 2. Materials and Methods

### 2.1. Cell Culture

The human breast cancer (BC) cell lines MDA-MB-231 and MDA-MB-468 were procured from ATCC (Manassas, VA, USA). Both cell lines were maintained in Dulbecco’s Modified Eagle Medium (DMEM) (GE Healthcare Life Sciences, Logan, UT, USA) in a humidified atmosphere of 5% CO_2_ at 37 °C. The medium was supplemented with 10% fetal bovine serum (FBS) (Atlanta Biologicals, Lawrenceville, GA, USA), penicillin (100 units/mL) and streptomycin (100 μg/mL) (Invitrogen, Carlsbad, CA, USA). Cells were intermittently tested for mycoplasma contamination at our institutional facility and determined to be free from mycoplasma.

### 2.2. Antibodies, microRNAs, and siRNAs

Antibodies, microRNAs, and siRNAs were purchased from the following sources: Anti–STAT3 (mouse monoclonal), pSTAT3-Y705 (rabbit monoclonal), and LIN28A (rabbit polyclonal) Cell Signaling Technology (Beverly, MA, USA). Anti-mouse and anti-rabbit horseradish peroxidase (HRP)-conjugated secondary antibodies from Santa Cruz Biotechnology (Santa Cruz, CA, USA). β-actin (mouse monoclonal) antibody from Sigma-Aldrich (St. Louis, MO, USA). Let-7a microRNA mimic and non-targeting control miRNA (miR-NC) from Thermo Fisher Scientific (Waltham, MA, USA). Non-targeting scrambled siRNAs (NT-Scr), or LIN28A targeting siRNAs, were from GE Healthcare Dharmacon, Inc. (Lafayette, CO, USA).

### 2.3. Treatment and Transfection

BC cells (MDA-MB-231 and MDA-MB-468) grown in 6- or 96-well plates were treated with resistin (Phoenix Pharma, Burlingame, CA, USA) as indicated in pertinent figure legends. For transient silencing of LIN28A or restoration of Let-7a, 60–70% confluent BC cells were transfected with NT-Scr/LIN28A targeted siRNAs, or 50 nM of miR-NC/Let-7a mimic using X-tremeGENE^TM^ HP DNA Transfection Reagent (Roche, Indianapolis, IN, USA) as per the manufacturer’s instructions.

### 2.4. Total RNA Isolation and Quantitative Reverse Transcription-Polymerase Chain Reaction (RT-PCR)

Total RNA from the BC cell lines treated with vehicle or resistin was extracted using the TRIzol reagent, and complementary DNA (cDNA) synthesized using 2 μg of total RNA. MicroRNA and mRNA expression analysis was performed using specific sets of primers (Table S1) by quantitative real-time PCR (qRT-PCR) as described previously [24]. GAPDH and U6 served as internal controls for mRNA and miRNA analysis, respectively. The thermal conditions for real-time PCR assays were as follows: cycle 1: 95 °C for 10 min, cycle 2 (40): 95 °C for 15 s, and 55–60 °C for 1 min.

### 2.5. Cell Growth Assay

MDA-MB-231 and MDA-MB-468 cells were transfected with miR-NC/Let-7a mimic or NT-Scr/LIN28A siRNA for 24 h. Then, cells were treated with resistin (20 ng/mL) for another 24 h. Subsequently, cells were trypsinized and seeded (1 × 10^5^) in 96-well plates, and the effect on growth was monitored by WST-1 assay (Roche Diagnostics, Mannheim, Germany) as performed previously [24].

### 2.6. Plating Efficiency Assay

For plating efficiency assay, cells were transfected with miR-NC/Let-7a mimic or NT-Scr/LIN28A siRNA for 24 h. Post transfection cells were treated with resistin for 24 h. Subsequently, cells were trypsinized and seeded (1 × 10^3^) in 6-well plates and allowed to grow for two weeks. Following two weeks of culture, colonies were fixed with methanol, stained with crystal violet, photographed, and counted using image analysis software (ImageJ, National Institutes of Health, Bethesda, MD, USA).

### 2.7. Sphere Formation Assay

BC cells were transfected with Let-7a mimic or LIN28A siRNA along with their respective controls for 24 h. Then, cells were treated with resistin for another 24 h. Subsequently, cells were harvested and seeded at a density of 1 × 10^3^/well in 6-well Ultra-Low attachment plates (Corning Incorporated, Corning, NY, USA) in a stem cell culture medium (DMEM: F-12K, 1:1; Life Technologies, Carlsbad, CA, USA) and allowed to develop tumorspheres. After two weeks of incubation, sphere development was counted and photographed using a phase-contrast microscope, as described previously [25].

### 2.8. Protein Extraction and Immunoblot Analyses

Cells were washed with 1X phosphate buffered saline (PBS) and lysed with NP-40 lysis buffer (Boston BioProducts, Ashland, MA, USA) supplemented with protease and phosphatase inhibitor cocktail (Thermo Fisher Scientific, Waltham, MA, USA). Next, samples were sonicated, centrifuged, and total protein was collected for further use. The protein samples (30 µg) were resolved on 10% SDS polyacrylamide gels and transferred to the PVDF membrane. The membrane was blocked with 5% milk for 1 h at room temperature and incubated with the specific primary antibodies overnight at 4 °C. Subsequently, blots were washed with 1× Tris-buffered saline with 0.1% Tween 20 (TBST) buffer and incubated for 1 h with horseradish peroxidase-conjugated secondary antibody. Protein signals were recorded using the West Femto Maximum Sensitivity Substrate kit (Thermo Scientific, Waltham, MA, USA) under Bio-Rad ChemiDoc Imager (Hercules, CA, USA). Furthermore, the uncropped Western blots were provided in Supplementary Figures S4–S9.

### 2.9. Ingenuity Pathway Analysis

Differentially expressed genes (fold change ±1.5 and *p*-value ≤ 0.05) from our targeted PCR array were subjected to the Ingenuity Pathway Analysis (IPA; Ingenuity Systems, Qiagen, http://www.ingenuity.com/products/ipa, accessed on 9 April 2019). ‘Upstream Analysis’ application of IPA was used to identify the upstream cascades driving observed changes in gene expression based on an activation z-score of ≥ |2|, as previously described [26].

### 2.10. Enzyme-Linked Immunosorbent Assay (ELISA)

MDA-MB-231 and MDA-MB-468 cells were transfected with Let-7a mimic or LIN28A siRNA along with their respective controls. After 24 h of transfection, cells were treated with resistin for another 48 h. Post-treatment cell culture supernatant was collected, centrifuged for 10 min at 2500 rpm at 4 °C to remove the cell debris, and the level of IL-6 was analyzed by human IL6 ELISA kit (D6050; R&D Systems, Minneapolis, MN, USA) as per manufacturer’s instructions.

### 2.11. Dual-Luciferase 3′ UTR-Reporter Assay

For the validation of STAT3 and IL-6 as a direct target of Let-7a, BC cells were transiently co-transfected for 24 h with 200 ng of STAT3 or IL-6 luciferase target-reporter plasmid containing STAT3 or IL-6 3′UTR region (GeneCopoeia, Rockville, MD, USA) and Renilla luciferase gene along with miRNA mimic control or Let-7a miRNA. In addition, we also generated mutants of STAT3, and IL-6 3′UTR (STAT3_MUT and IL-6_MUT) reporter construct by site-directed mutagenesis in the putative target region of Let-7a using Quickchange XL site-directed mutagenesis kit (Agilent Technologies, Santa Clara, CA, USA) and transiently transfected BC cells as described above. After that, BC cells were treated with control or resistin for 24 h, and cells were harvested in reporter lysis buffer (Promega Corporation, Madison, WI, USA). Luciferase activities were measured using a dual-luciferase assay kit (Promega Corporation) according to the manufacturer’s instructions.

### 2.12. Site-Directed Mutagenesis

Site-directed mutagenesis was performed using a QuickChange Multi site-directed mutagenesis kit (Agilent Technologies) according to the manufacturer’s instructions. The STAT3 and IL-6 3′UTR luciferase reporter vectors were used as the templates. In the STAT3 3′UTR plasmid, Let-7a binding site sequence 5′ CUACCUC 3′ was mutated to 5′ CUGCUUC 3′, and in IL-6 3′UTR plasmid, Let-7a binding site sequence 5′ UACCUC 3′ was mutated to 5′ UGCUUA 3′ by PCR using specific sets of primers (Table S1). The plasmids were isolated using the Qiagen Miniprep Kit (Qiagen Inc. Germantown, MD, USA), and DNA sequencing (Eurofins, Louisville, KY, USA) was performed to confirm the mutations.

### 2.13. Statistical Analysis

The experiments were performed at least three times with three technical replicates, and data expressed as mean ± SD wherever appropriate. In addition, the data were subjected to an unpaired two-tailed Student’s *t*-test for comparative analyses. A *p*-value of < 0.05 was considered statistically significant.

## 3. Results

### 3.1. Resistin Downregulates the Expression of Let-7a in Breast Cancer Cells

**Figure 1:**
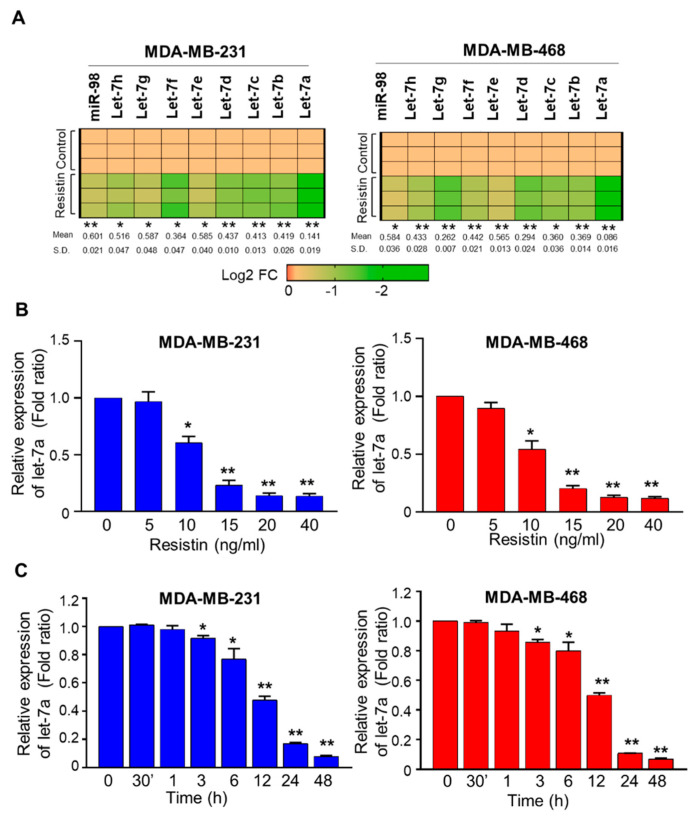
Effect of resistin on Let-7a expression. (**A**) MDA-MB-231 and MDA-MB-468 BC cells were treated with resistin, and the expression of Let-7 family miRNAs was analyzed by qRT-PCR. (**B**) Dose-dependent regulation of Let-7a by resistin (0–40 ng/mL) examined after 24 h. (**C**) Time-course of Let-7a downregulation following treatment with resistin (20 ng/mL). Error bars represent the mean ± SD; *n* = 3, * *p* < 0.05, ** *p* < 0.001. U6 was used as an internal control for the analyses.

To investigate the effect of resistin on the expression of the Let-7 family of miRNAs, we treated two BC cell lines, MDA-MB-231 and MDA-MB-468, with resistin (20 ng/mL) for 24 h and the expression of Let-7 miRNAs was examined. A repressive effect of resistin was observed on all Let7 family miRNAs; however, the most noticeable and significant downregulation was observed for Let7a in both the cell lines (Figure 1A). We next treated the BC cells with varying doses of resistin for 24 h to observe a dose-response on Let-7a expression. A dose-dependent downregulation of Let-7a following resistin treatment was observed in both cell lines, with significant differences recorded at 10–40 ng/mL doses (Figure 1B). Similarly, we also conducted a time-course study (0–48 h) to study resistin-mediated Let-7a regulation. The data show a noticeable and significant decrease in the expression of Let-7a by 3 h that continues to decrease further with nearly 12.6- and 14.2-fold downregulation in MB-231 and MB-468 cells, respectively, at 48 h (Figure 1C). Notably, resistin treatment did not alter the expression of pri-miRNA transcripts of Let-7a family members (Let-7a-1, Let-7a-2, and Let-7a-3) (Figure S1). Thus, our data suggest that resistin induces a dose- and time-dependent decrease in Let-7a expression in BC cells.

### 3.2. Suppression of Let-7a by Resistin in Breast Cancer Cells Is Mediated through LIN28A

**Figure 2:**
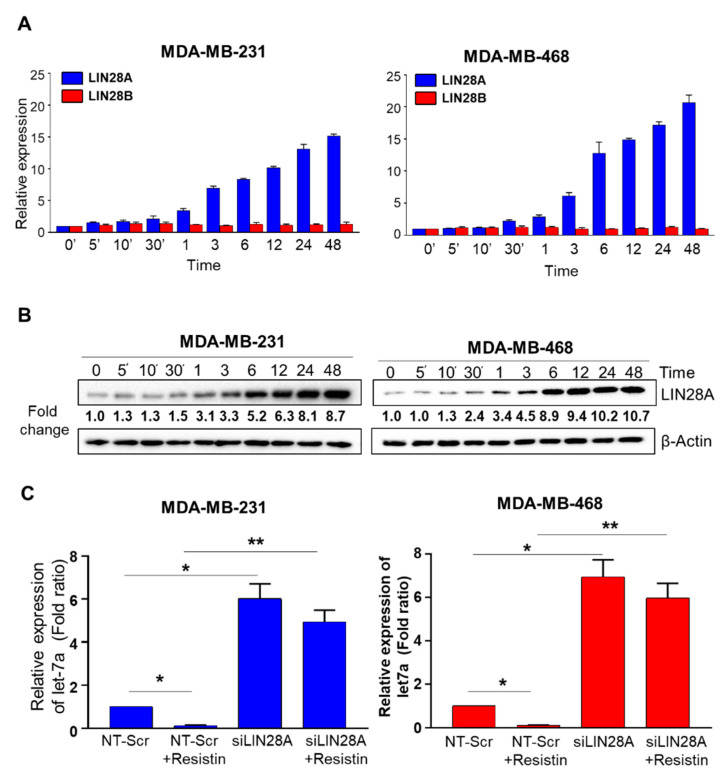
Resistin-induced Let-7a downregulation is mediated through LIN28A in breast cancer cells. BC cells were grown in a 6-well plate and treated with 20 ng/mL resistin for indicated time intervals, and the expression of LIN28A was examined at mRNA level by quantitative RT-PCR (**A**) and at the protein level by immunoblot assay (**B**). GAPDH (for mRNA) and β-actin (for protein) were used as internal controls. (**C**) BC cells were grown in six-well plates and transfected with NT-Scr or LIN28A-targeting siRNAs. After 24 h of transfection, cells were treated with resistin (20 ng/mL) for 24 h, RNA was isolated, and expression of Let-7a was monitored by RT-PCR. U6 was used as an internal control. Data are presented as mean ± S.D. *n* = 3. * *p* < 0.05, ** *p* < 0.001.

Having observed that resistin led to a decreased expression of matured Let-7a but not its precursor forms, we sought to examine the involvement of LIN28A and LIN28B, the two highly related RNA-binding proteins known to regulate the maturation of Let-7 family miRNAs [14,17]. BC cells were treated with resistin (20 ng/mL) for different time durations ranging from 5 min to 48 h, and the expression of LIN28A and LIN28B was examined by quantitative RT-PCR. We observed that LIN28A expression increased in a time-dependent manner in both the BC cell lines, while LIN28B levels remained largely unaltered (Figure 2A). We further confirmed the expression of LIN28A at the protein level by immunoblot analysis. A similar time-dependent increase in LIN28A expression was reported, with noticeable differences seen as early as 1 h. (Figure 2B). After that, we examined if resistin-induced LIN28a upregulation was associated with Let7a downregulation. For this, we transiently silenced the expression of LIN28a using specific siRNAs. By 24 h, we observed over 80–90% decrease in LIN28A expression that persisted for at least 72 h (Figure S2). Therefore, we first treated the BC cells with LIN28A-specific siRNAs for 24 h and then incubated them with resistin for next 24 h. After that, we measured the levels of Let7a by qRT-PCR. The data show that pre-silencing of LIN28A not only abrogated resistin-induced suppression of Let-7a but instead led to its significant upregulation over control (NT-Scr) treated cells (Figure 2C). These findings thus suggest that LIN28A mediates basal as well as resistin-induced repression of Let-7a in BC cells.

### 3.3. Let-7a Restoration or Silencing of LIN28A Abrogates Resistin-Induced Growth, Clonogenic Survival, and Sphere-Forming Ability of Breast Cancer

**Figure 3:**
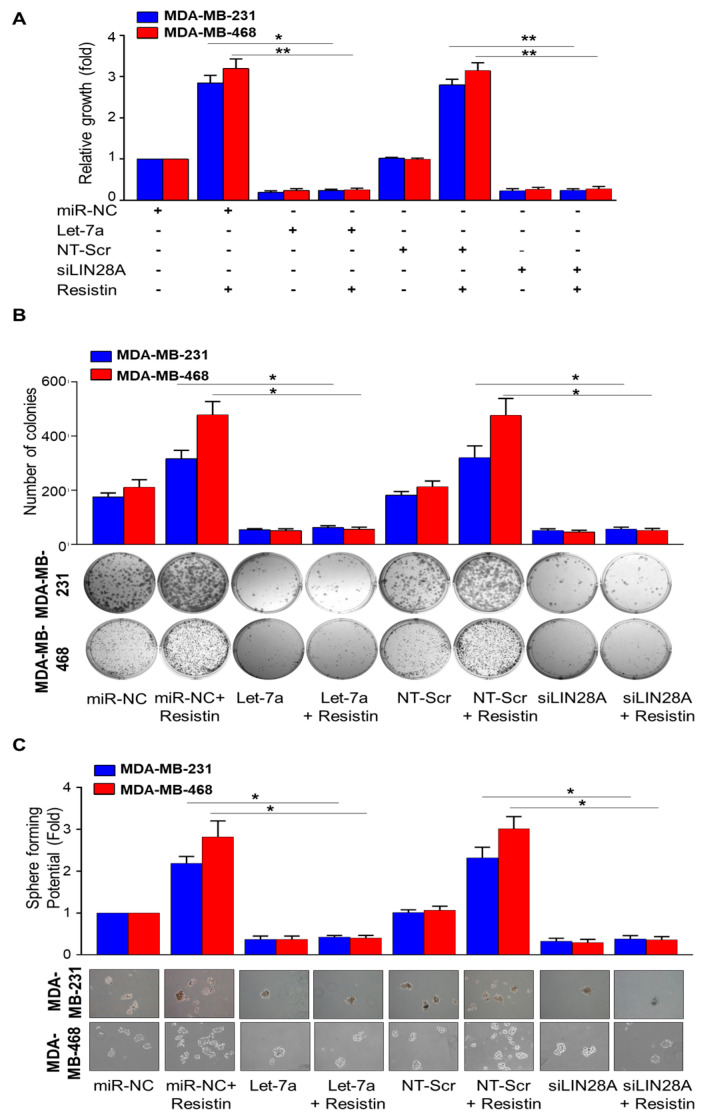
LIN28A/Let-7-miR axis mediates resistin-induced growth and stemness of breast cancer cells. (**A**) MDA-MB-231 and MDA-MB-468 BC cells were transfected with NT-Scr/miR Control (miR-NC) or siLin28A/Let-7a mimics and treated with resistin (20 ng/mL). After 24 h, cells were trypsinized and seeded in 96-well plates. Growth was measured after 96 h by WST-1 assay. (**B**) BC cells were transfected with NT-Scr/miR-NC or siLin28A/Let-7a mimic. After transfection, BC cells were treated with resistin for 24 h, trypsinized, counted, and seeded at low density (1 × 10^3^ cells per well) in 6-well plates. After two weeks, colonies were stained with crystal violet and visualized and photographed using the imaging system. (**C**) BC cells transfected with Let-7a or LIN28A, treated with resistin, and seeded at low density (1 × 10^3^) in ultra-low attachment plates and allowed to grow for two weeks in stem cell culture medium. Images were taken under a light microscope at 200× magnification, and the number of spheres was counted manually. Data represents mean ± S.D. *n* = 3, * *p* < 0.05, ** *p* < 0.001.

Earlier, we demonstrated that resistin promoted the growth, aggressiveness, and sphere-forming ability of BC cells [19,23]. Therefore, considering the role of resistin in the regulation of Let-7a and LIN28A, we analyzed the effect of Let-7a restoration and LIN28A silencing on resistin-induced BC phenotypes. For this, we transfected the BC cells with Let-7a mimic or LIN28A specific siRNAs for 24 h along with their respective controls before treatment with resistin. After that, the effect on growth, clonogenicity, and sphere-forming ability on ultra-low attachment plates was examined. Our data show that the treatment with Let-7a mimics or LIN28A siRNAs significantly inhibited the growth of MB-231 (~5.0 fold and ~4.4 fold, respectively) and MB-468 (~4.1 fold and ~3.7 fold, respectively) cells. Furthermore, Let-7a mimic or LIN28A siRNA-pretreated BC cells do not exhibit significantly increased growth when treated with resistin (Figure 3A). Similarly, we observe that restoration of Let-7a or LIN28A silencing significantly inhibits the colony-forming and sphere-forming ability of BC cells (Figure 3B,C). Moreover, resistin-induced colony and mammosphere formation is also abrogated in BC cells that had been transfected with Let-7a mimic or LIN28A siRNAs (Figure 3B,C).

### 3.4. Let-7a Downregulation Caused by Resistin Treatment Is Associated with Upregulation of STAT3 and IL-6 in Breast Cancer Cells

**Figure 4:**
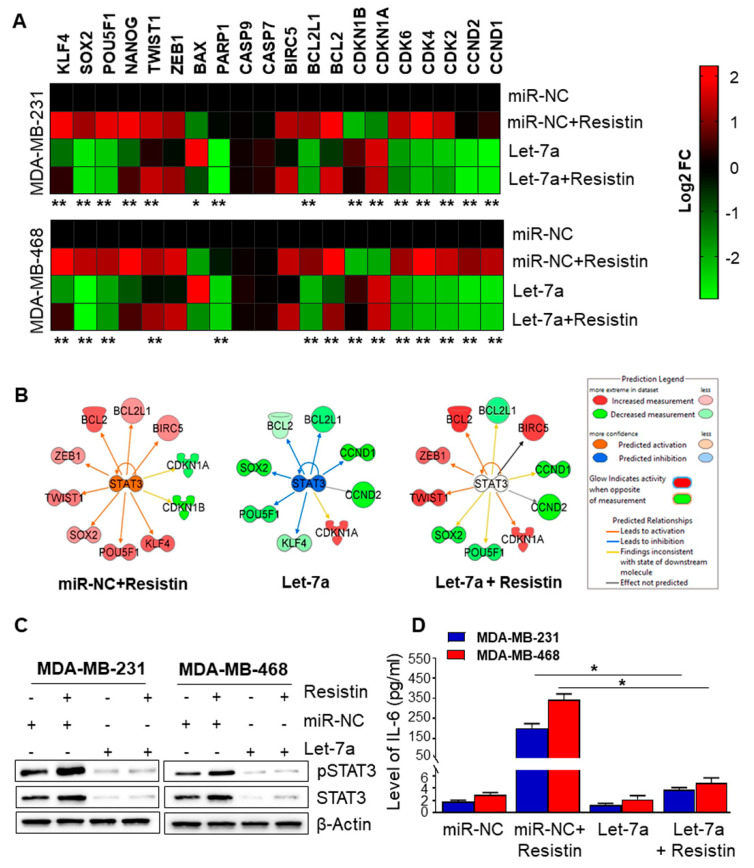
STAT3 activation is associated with resistin-induced, Let-7a-mediated effects on gene expression. (**A**) MDA-MB-231 and MDA-MB-468 cells were transfected with miR-NC or Let-7a mimic for 24 h, treated with resistin (20 ng/mL), and expression of growth, survival, and stemness related genes were analyzed by qRT-PCR. GAPDH was used as an internal control. (**B**) Ingenuity Pathways Analysis was performed to interpret the gene regulatory networks in BC cells after treatment with resistin or Let-7a. (**C**) BC cells were transfected with Let-7a mimic for 24 h, treated with resistin, and the expression of pSTAT3 and STAT3 was analyzed by western blot. β-actin was used as an internal control. (**D**) BC cells were transfected with Let-7a mimic for 24 h, and subsequently treated with resistin, and the supernatant was collected after 48 h. Levels of IL-6 were measured using ELISA assay. Data represent mean ± S.D. *n* = 3. * *p* < 0.05, ** *p* < 0.005.

Having observed the involvement of Let-7a in resistin-induced growth, survival, and stemness potential of BC cells, we next sought out to examine the specific target(s) of Let-7a in BC cells. For this, we analyzed the expression of genes involved in cell growth, survival, and stemness by RT-PCR analyses using focused arrays in resistin and/or Let-7 mimic-treated BC cells. We observed that resistin altered the expression of several genes associated with cell survival, cell cycle, and stemness in BC cells, and pre-treatment with Let-7a mimics abrogated resistin-induced changes in gene expression (Figure 4A). Next, we performed the Ingenuity Pathways Analysis (IPA) to identify the gene regulatory networks associated with observed altered gene expression in BC cells after Let-7a restoration and/or resistin treatment. Our analysis revealed the Stat3 signaling as the most prominent regulatory pathway associated with resistin and Let-7a mimic treatment-induced gene expression changes (Figure 4B). Moreover, during in silico analysis, TargetScan also identified STAT3 as a putative Let-7a gene target (Figure S3A). Interestingly, we also identified IL6 as a putative target of Let-7a (Figure S3B). Therefore, we examined the effect of Let-7a restoration on Stat3 and IL-6. BC cells were transfected with either miRNA control or Let-7a for 24 h and then treated with resistin for 48 h. The impact on the expression of STAT3 and pSTAT3 was analyzed by immunoblotting, and that of IL6 was analyzed by ELISA. Our data show increased levels of total and phospho-STAT3 and IL6 in resistin-treated BC cells (Figure 4C,D). Moreover, resistin-induced expression of STAT3 and IL6 was abrogated in cells pretreated with Let-7a mimics (Figure 4C,D).

### 3.5. Let-7a Suppresses Resistin-Induced STAT3 and IL-6 Expression by Directly Targeting Its 3′UTR

**Figure 5:**
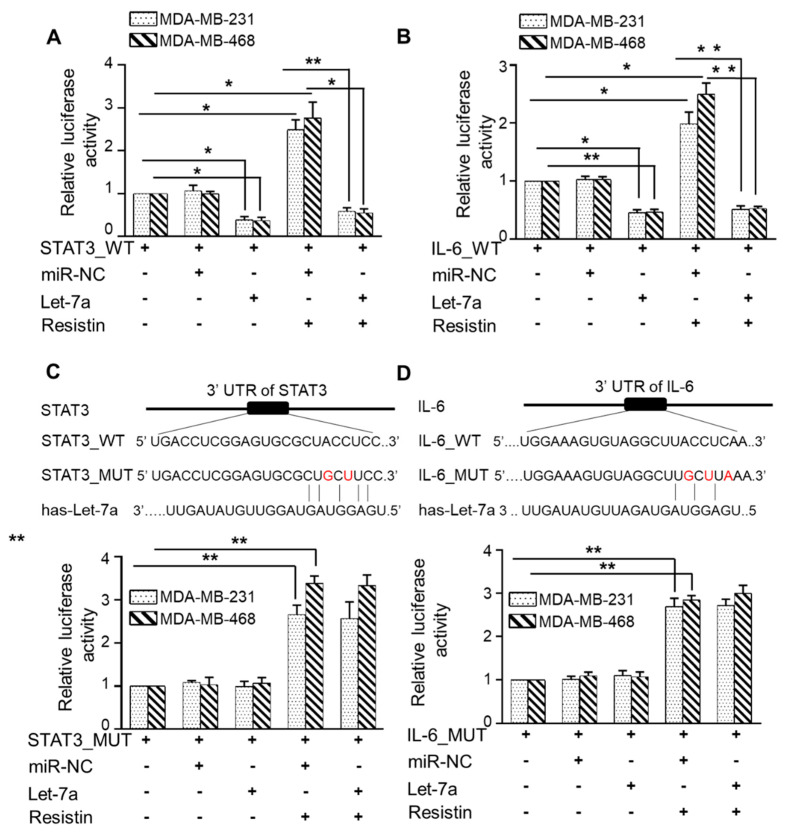
Let-7a suppresses STAT3 and IL6 expression in breast cancer cells by directly targeting their 3′ untranslated regions. MDA-MB-231 and MDA-MB-468 cells were grown in 6-well plates and transiently co-transfected with luciferase reporter plasmids containing either STAT3 3′UTR (**A**) or IL6 3′UTR (**B**) and miR control (miR-NC) or Let-7a. Following 24 h of transfection, cells were treated with resistin for the next 24 h, and total protein lysates were made in the passive lysis buffer. Luciferase activities were measured using a dual-luciferase assay system. To ascertain that the effect of Let-7a on luciferase activity was due to its direct binding, we mutated 3′ UTRs of STAT3 (**C**) and IL6 (**D**) as indicated. Data (mean ± SD.; *n* = 3, **, *p* < 0.005) is presented as fold change in normalized luciferase activity. * *p* < 0.05, ** *p* < 0.005.

To experimentally validate direct targeting of STAT3 and IL6 by Let-7a, we transiently co-transfected the BC cells with STAT3 or IL-6 3′UTR luciferase reporter plasmids with Let-7a mimic, and luciferase activity was measured after resistin treatment. We observed that resistin treatment significantly increased the luciferase activity in STAT3 (Figure 5A) and IL-6 (Figure 5B) 3′UTR-Reporter plasmid transfected MDA-MB-231 (~2.4 fold and ~1.9 fold, respectively) and MDA-MB-468 (~2.7 fold and 2.4 fold, respectively) cells. Furthermore, transfection of Let-7a mimics significantly reduced the luciferase activity in STAT3 and IL-6 3′UTR-Reporter transfected BC cells (Figure 5A,B). To confirm if this downregulation of luciferase reporter activity resulted from direct binding of Let-7a on the 3′UTR, we mutated these regions in STAT3 and IL-6 3′UTR-Reporter plasmids and used these mutant plasmids in reporter assays (Figure 5C,D). The data revealed that BC cells transfected with mutated-3′UTR of STAT3 or IL-6 did not show any response to the suppressor activity of Let-7a. Altogether, our data suggest that resistin-induced downregulation of Let-7a is associated with overexpression of STAT3 and IL-6 expression in BC cells.

## 4. Discussion

Breast cancer (BC) remains the second leading cause of cancer-related death in women in the United States, exhibiting significant racial disparities in the age of onset, disease aggressiveness, and clinical outcomes [27,28]. Root causes of race-associated health disparities remain mostly unknown, but the possible risk factors include socioeconomic differences, differential access to health care, biological and non-biological differences, and disease-related molecular mechanistic differences [2,20,27,28]. Our earlier work demonstrated a significantly increased expression of resistin, Stat3, and IL6 in AA women’s serum with BC than CA patients and associated with enhanced BC cell growth, aggressiveness, stemness, and therapy resistance [19,23]. In this study, we have provided the data to demonstrate a novel resistin-LIN28A-Let-7a-STAT3/IL-6 mechanistic link supporting the growth and stemness of BC cells.

Let-7a is a member of the Let-7 family, whose dysregulation has long been associated with onset and disease progression, prognosis, and therapy resistance in BC patients [15,16,29]. Out of the nine Let-7 family members, we observed the most downregulation of Let-7a in resistin-treated BC cells. Furthermore, we observed a more significant downregulation of Let-7a in AA BC cells than CA BC cells. It is intriguing and could be associated with increased expression of CAP1, a resistin receptor, in AA BC cells [19]. The lower expression of Let-7a has been reported in BC tissues than the adjacent normal tissues [30]. Furthermore, reduced expression of Let-7a is reported in HER2+ breast tumors compared to HER2-, and is inversely associated with the expression of DNA repair protein, PARP1 [31]. Since we used triple-negative BC cell lines in our study, resistin-mediated downregulation of Let-7a may represent another mode through which Let-7a can be regulated in cancer cells. Our findings also suggest a need for more studies to explore the extent and association of Let-7a expression with breast tumor subtypes.

We also demonstrated, for the first time, resistin-mediated upregulation of LIN28A in BC cells. LIN28A is an RNA-binding protein that is overexpressed in many cancers [32,33]. Furthermore, overexpression of LIN28A and/or its related protein LIN28B is also associated with the prognosis of patients [34]. An earlier study demonstrated that LIN28A was abundantly expressed in androgen receptor-positive BC, and their coexpression positively associated with tumor grade and poor prognosis [35]. Further, LIN28-mediated Let7a downregulation is suggested to be an important mechanism for context-dependent alterations in the expression of the Let7 family [11,36]. The differential regulation of Let-7 by LIN28A and LIN28B is attributed to their distinct subcellular localization. LIN28A primarily localizes to the cell cytoplasm, whereas LIN28B contains nuclear localization signals and specifically localizes to the nucleus [14]. LIN28A is shown to bind the terminal loop of pre-Let-7 by recruiting terminal uridylyltransferase, Zcchc11, leading to its degradation or blocking the miRNA maturation [13,14,36,37], whereas LIN28B binds to primary-Let-7 transcript and inhibit its processing in a Zcchc11-independent manner. In other reports, c-MYC, SOX2, and NF-κB have been shown to regulate LIN28 expression in different model systems, including cancer [38,39,40]. Moreover, an earlier report has also demonstrated that resistin activates NF-κB to stimulate pro-inflammatory cytokines, TNF-α, and IL-12 [41]. Thus, it will be interesting to investigate a broader impact of resistin in terms of altering the secretome of BC cells in future studies.

Our study established that LIN28A-mediated Let-7a downregulation was involved in resistin-induced growth, clonogenicity, and stemness in BC cells. The efficacy of many frontline therapies is compromised due to their inability to kill cancer stem cells (CSC), leading to tumor relapse, metastasis, and therapy resistance [42,43,44]. We earlier demonstrated a role of resistin in potentiating the stemness of BC cells, as was evident by induced expression of CD44, ALDH, and a more remarkable sphere-forming ability [23]. LIN28A is also shown to play a vital role in CSCs, leading to tumor aggressiveness, metastasis, and therapy resistance [36,45]. In other reports, Let-7a is shown to effectively repress the capability of sphere formation in hepatic cancer cells by regulating the Wnt signaling pathway [46]. Additionally, lower Let-7a expression is associated with therapy resistance in HER2^+^ primary breast tumors [47]. Moreover, the upregulation of Let7a expression sensitizes resistant BC cells to chemosensitivity by enhancing apoptosis [47]. Thus, our findings establishing resistin-mediated downregulation of Let7a in BC and its functional significance reveal novel nodes for therapeutic intervention to manage the aggressive and hard-to-treat disease subtype.

STAT3 is aberrantly hyperactivated in many human malignancies, including BC, and such hyperactivation is generally associated with inadequate therapeutic response to chemotherapy [48,49]. It is also emerging as a clinically useful target in triple-negative BC [49]. STAT3 regulates a variety of important genes involved in aggressive tumor behavior, stem cell properties, and cancer chemoresistance [47,49]. IL-6 is known to drive STAT3 phosphorylation via receptor-associated Janus Kinases, and activation of the IL-6/STAT3 signaling pathway is reported in several inflammation-associated human malignancies, including BC [50]. Interestingly, we earlier reported that resistin treatment of BC cells led to both enhanced expression and phosphorylation of STAT3 [19]. Our current findings further these observations and unveil a novel mechanism for resistin-mediated regulation of STAT3 expression and phosphorylation via IL6. Importantly, our findings linking resistin with LIN28A/Let-7a signaling axis are highly significant and add to our understanding of breast tumor biology.

## 5. Conclusions

**Figure 6:**
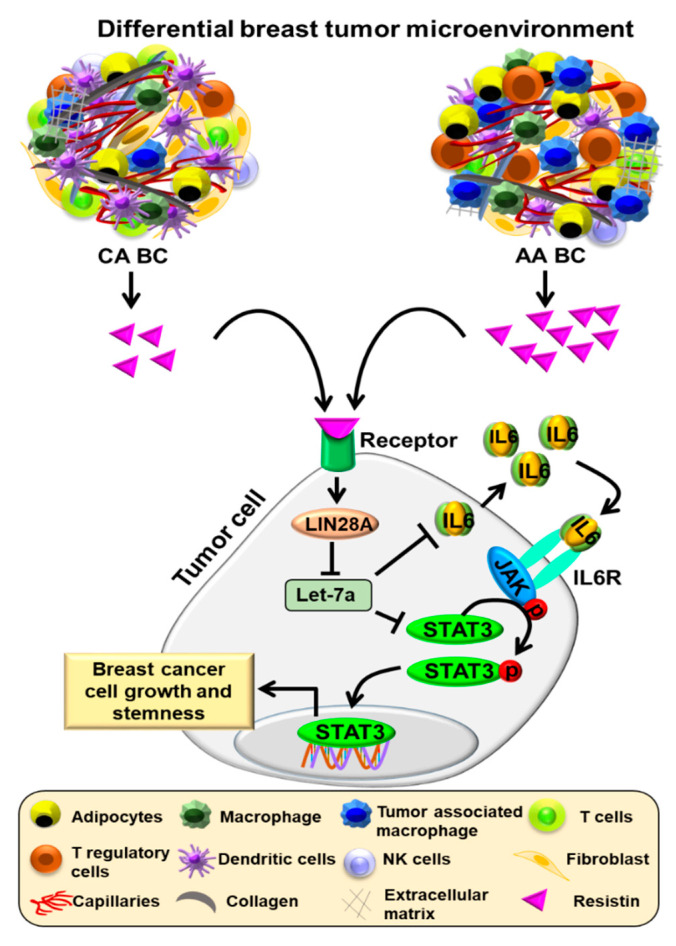
A schematic showing Let7a-mediated effects of resistin in breast cancer cells. Racially disparate tumor microenvironment in breast tumors leads to the release of higher levels of resistin in African American BC patients than CA patients. Resistin increases the expression of LIN28A, which then represses the maturation of Let-7a miRNA leading to enhanced expression of IL-6 and STAT3/pSTAT3. Activated STAT3 alters the expression of genes associated with the growth and stemness potential of BC cells.

Taken together, we demonstrate a novel resistin-LIN28A-Let-7a-STAT3/IL-6 signaling loop supporting the growth, clonogenicity, and stemness of BC cells (Figure 6). It will be of interest to study the broader significance of these findings and examine if resistin exerts similar effects in other malignancies. Further, exploring the clinical relevance of these findings will provide additional data to support the importance of this signaling axis and pave the way for improved clinical management of BC and race-associated disparate clinical outcomes.

## Data Availability

All the data presented in this article are available from the corresponding author on reasonable request.

## Supplementary Materials

The following are available online at https://www.mdpi.com/article/10.3390/cancers13184498/s1, Figure S1: Effect of resistin on Let-7a pri-miRNA transcripts expression, Figure S2: Silencing of LIN28A in breast cancer cells, Figure S3: In silico analysis (using algorithms of TargetScan) showing Let-7a-binding sites in STAT3 and IL-6 3′UTR, Figures S4–S9: Uncropped Western blot images, Table S1: List of primers used in this study.
