# Supplementary material for: Resistin Induces LIN28A-Mediated Let-7a Repression in Breast Cancer Cells Leading to IL-6 and STAT3 Upregulation
Source: Cancers (Basel). 2021 Sep 7;13(18):4498. doi: 10.3390/cancers13184498 (PMC8470467; doi:10.3390/cancers13184498)
Supplement: Supplementary file 1 [file cancers-13-04498-s001.zip › cancers-1342881-supplementary.pdf]

cancers

MDPI

Supplementary Materials: Resistin Induces LIN28A-Mediated Let-7a Repression in Breast Cancer Cells Leading to IL-6 and STAT3 Upregulation

Sachin Kumar Deshmukh, Sanjeev Kumar Srivastava, Haseeb Zubair, Mohammad Aslam Khan, Ajay Pratap Singh and Seema Singh

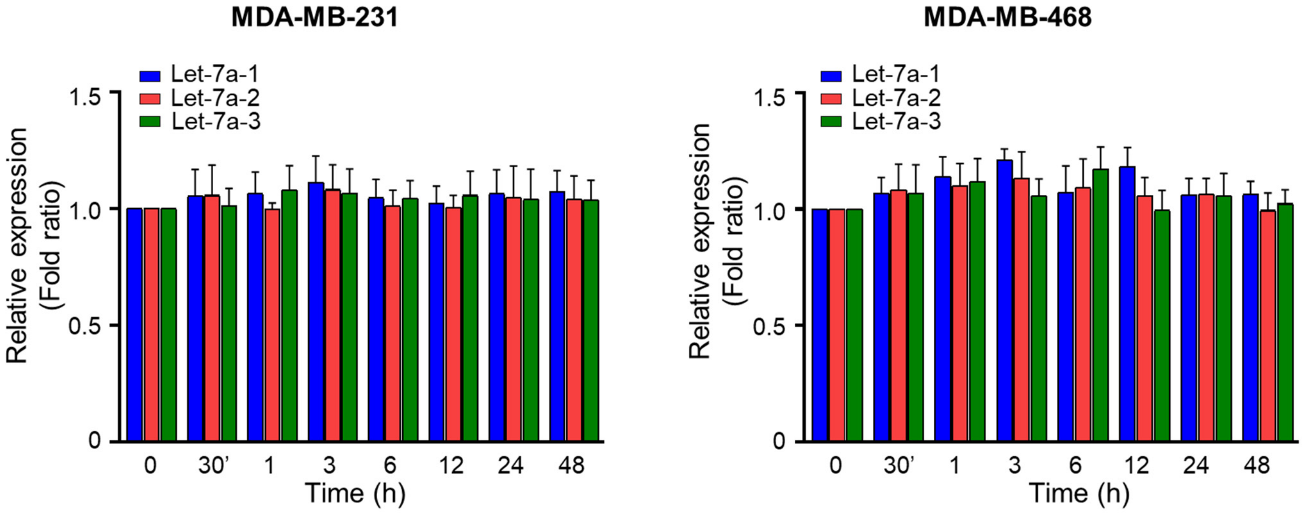

Figure S1. Effect of resistin on Let-7a pri-miRNA transcripts expression. MDA-MB-231 and MDA-MB-468 BC cells were grown in a 6-well plates were treated with resistin (0–20 ng/mL) for different time intervals (0–48 h). RNA was isolated and the expression of pri-miRNA transcripts of Let-7a family members Let-7a-1, Let-7a-2, and Let-7a-3 were analyzed by RT-PCR. RNU48 was used as an internal control. Bars represent the mean ± SD; $n = 3$.

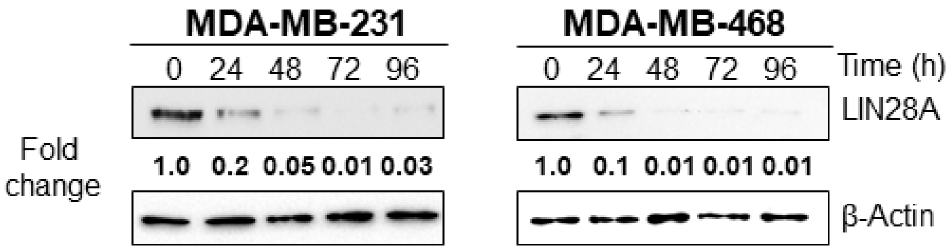

Figure S2. Silencing of LIN28A in breast cancer cells. MDA-MB-231 and MDA-MB-468 BC cells were transfected with LIN28A-specific siRNA and total protein was extracted at different time intervals (0–96 h). Immunoblot assay was performed to measure changes in LIN28A expression levels. β-actin was used as an internal control.

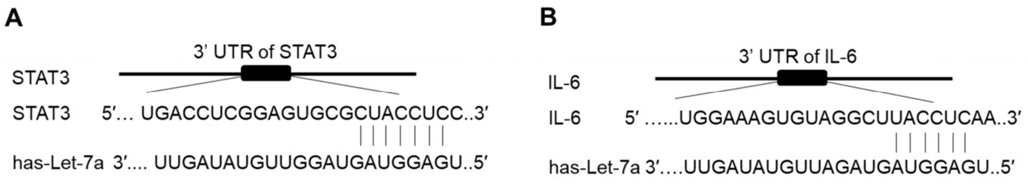

Figure S3. In silico analysis (using algorithms of TargetScan) showing Let-7a-binding sites in STAT3 **(A)** and IL-6 **(B)** 3′UTR.

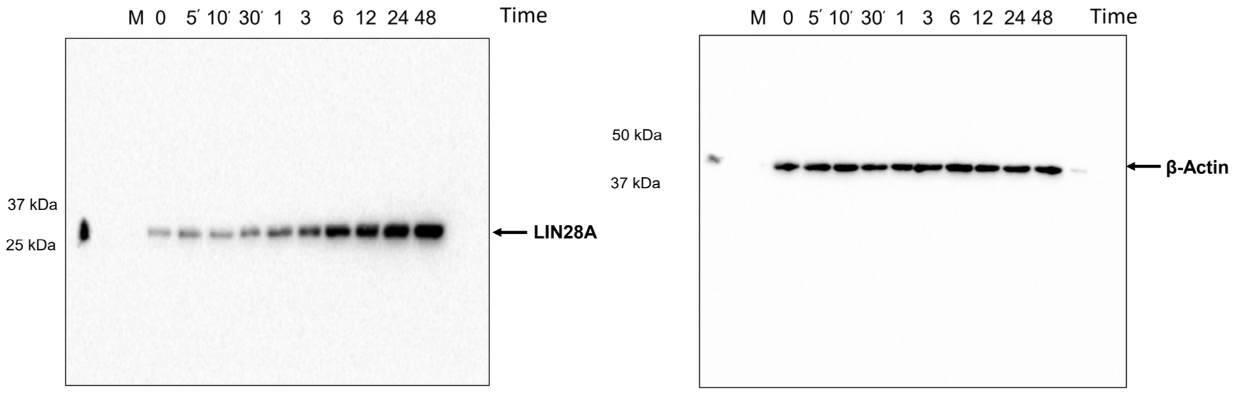

Figure S4. Resistin-induced Let-7a downregulation is mediated through LIN28A in breast cancer cells. MDAMB-231 breast cancer cells were grown in a 6-wells plate and treated with 20 ng/mL resistin for indicated time intervals, and the expression of LIN28A was examined at the protein level by immunoblot assay. β-actin were used as internal control.

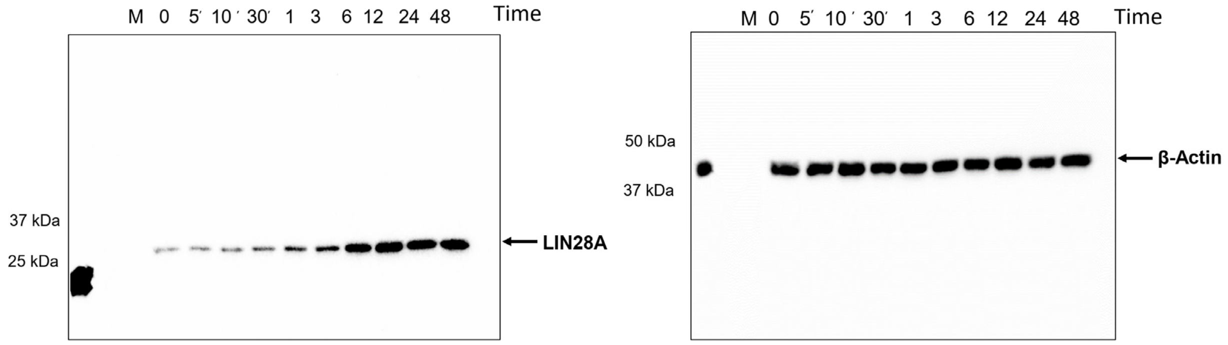

Figure S5. Resistin-induced Let-7a downregulation is mediated through LIN28A in breast cancer cells. MDAMB-468 breast cancer cells were grown in a 6-wells plate and treated with 20 ng/mL resistin for indicated time intervals, and the expression of LIN28A was examined at the protein level by immunoblot assay. β-actin were used as internal control.

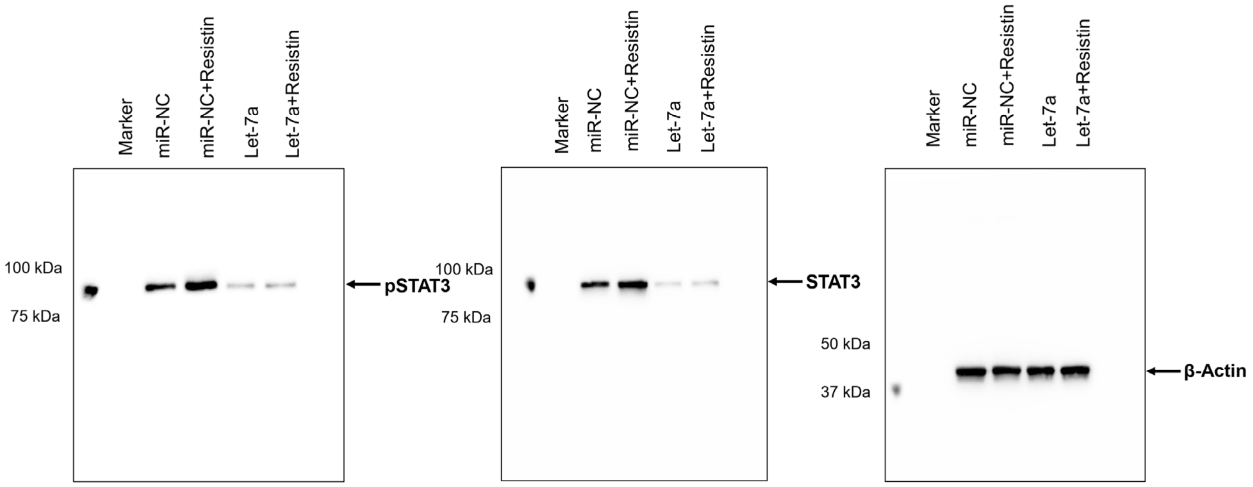

Figure S6. STAT3 activation is associated with resistin-induced, Let-7a-mediated effects on gene expression. MDA-MB-231 breast cancer cells were transfected with Let-7a mimic for 24 h, treated with resistin, and the expression of pSTAT3 and STAT3 was analyzed by western blot. β-actin was used as an internal control.

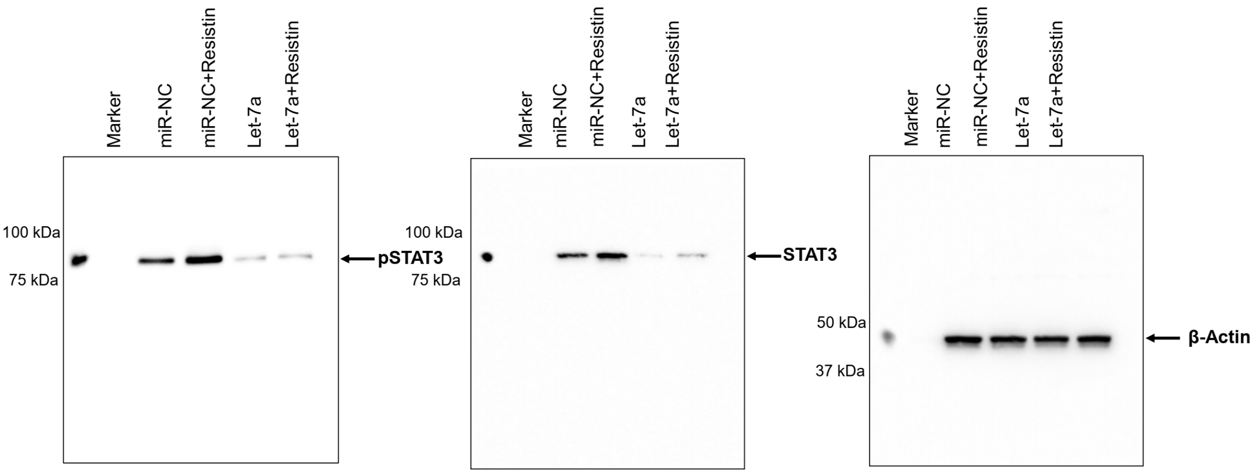

Figure S7. STAT3 activation is associated with resistin-induced, Let-7a-mediated effects on gene expression. MDA-MB-468 breast cancer cells were transfected with Let-7a mimic for 24 h, treated with resistin, and the expression of pSTAT3 and STAT3 was analyzed by western blot. β-actin was used as an internal control.

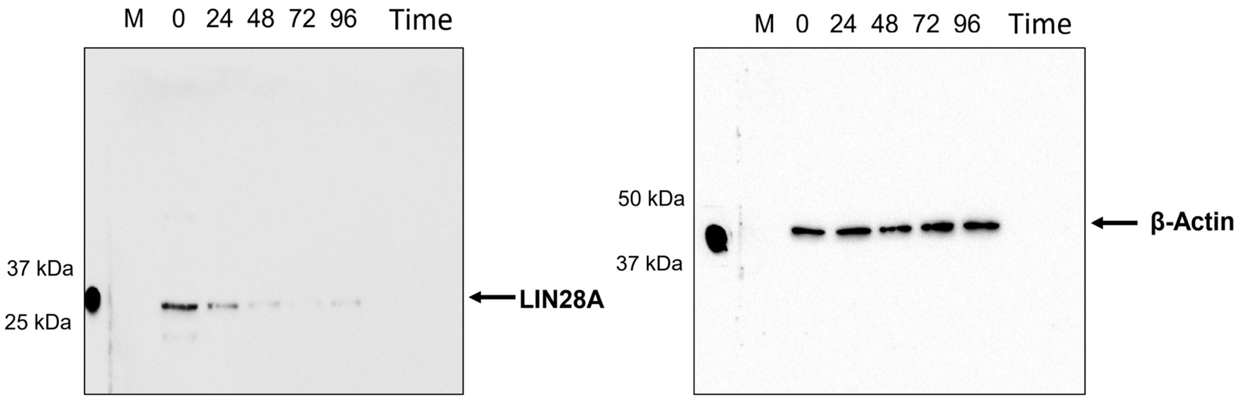

Figure S8. Silencing of LIN28A in MDA-MB-231 breast cancer cells. MDA-MB-231 breast cancer cells were transfected with LIN28A-specific siRNA and total protein was extracted at different time intervals (0–96 h). Immunoblot assay was performed to measure changes in LIN28A expression levels. β-actin was used as an internal control.

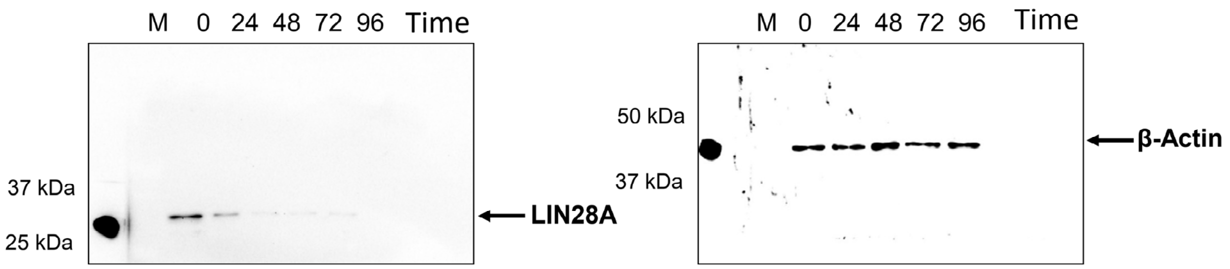

Figure S9. Silencing of LIN28A in MDA-MB-468 breast cancer cells. MDA-MB-468 breast cancer cells were transfected with LIN28A-specific siRNA and total protein was extracted at different time intervals (0–96 h). Immunoblot assay was performed to measure changes in LIN28A expression levels. β-actin was used as an internal control.

Table S1. List of primers used in this study.

S. No.	Primer	Sequence (5′-3′)
1	Let-7a	RT: GTCGTATCCAGTGCAGGGTCCGAGGTATTCGCACTGGATACGACAACTAT<br>Forward: TCGGCGTGAGGTAGTAGGTTGT
2	Let-7b	RT: GTCGTATCCAGTGCAGGGTCCGAGGTATTCGCACTGGATACGACAACCAC<br>Forward: TCGGCGTGAGGTAGTAGGTTGT
3	Let-7c	RT: GTCGTATCCAGTGCAGGGTCCGAGGTATTCGCACTGGATACGACAACCAT

		Forward: TCGGCGTGAGGTAGTAGGTTGT
4	Let-7d	RT: GTCGTATCCAGTGCAGGGTCCGAGGTATTCGCACTGGATACGACAACTAT<br>Forward: TCGGCGAGAGGTAGTAGGTTGC
5	Let-7e	RT: GTCGTATCCAGTGCAGGGTCCGAGGTATTCGCACTGGATACGACAACTAT<br>Forward: TCGGCGTGAGGTAGGAGGTTGT
6	Let-7f	RT: GTCGTATCCAGTGCAGGGTCCGAGGTATTCGCACTGGATACGACAACTAT<br>Forward: TCGGCGTGAGGTAGTAGATTGT
7	Let-7g	RT: GTCGTATCCAGTGCAGGGTCCGAGGTATTCGCACTGGATACGACAACTGT<br>Forward: TCGGCGTGAGGTAGTAGTTTGT
8	Let-7i	RT: GTCGTATCCAGTGCAGGGTCCGAGGTATTCGCACTGGATACGACAACAGC<br>Forward: TCGGCGTGAGGTAGTAGTTTGT
9	miR-98	RT: GTCGTATCCAGTGCAGGGTCCGAGGTATTCGCACTGGATACGACAACAAT<br>Forward: TCGGCGTGAGGTAGTAAGTTGT
10	miR-17	RT: GTCGTATCCAGTGCAGGGTCCGAGGTATTCGCACTGGATACGACCTACCT<br>Forward: TCGGCGCAAAGTGCTTACAGTGC
11	Universal	Reverse: GTCGTATCCAGTGCAGGGTCCGAGGT
12	U6	RT: AAAATATGGAACGCTTCACGAATTTG<br>Forward: CTCGCTTCGGCAGCACATATACT<br>Reverse: ACGCTTCACGAATTTGCGTGTC
13	KLF4	Forward: GAAATTCGCCCGCTCCGATGA<br>Reverse: CTGTGTGTTTGCGGTAGTGCC
14	SOX2	Forward: GCCGAGTGGAAACTTTTGTCG<br>Reverse: GGCAGCGTGTACTTATCCTTCT
15	POU5F1	Forward: CTTGAATCCCGAATGGAAAGGG<br>Reverse: GTGTATATCCCAGGGTGATCCTC
16	NANOG	Forward: TTTGTGGGCCTGAAGAAAACT<br>Reverse: AGGGCTGTCCTGAATAAGCAG
17	TWIST1	Forward: GCCAGGTACATCGACTTCCTCT<br>Reverse: TCCATCCTCCAGACCGAGAAGG
18	ZEB1	Forward: TTACACCTTTGCATACAGAACCC<br>Reverse: TTTACGATTACACCCAGACTGC
19	BAX	Forward: CCCGAGAGGTCTTTTTCCGAG<br>Reverse: CCAGCCCATGATGGTTCTGAT
20	PARP1	Forward: CGGAGTCTTCGGATAAGCTCT<br>Reverse: TTTCCATCAAACATGGGCGAC
21	CASP7	Forward: CGGTCCTCGTTTGTACCGTC<br>Reverse: CGCCCATACCTGTCACTTTATCA
22	CASP9	Forward: CTCAGACCAGAGATTCGCAAAC<br>Reverse: GCATTTCCCCTCAAACTCTCAA
23	BIRC5	Forward: AGGACCACCGCATCTCTACAT<br>Reverse: AAGTCTGGCTCGTTCTCAGTG
24	BCL2L1	Forward: GCCACTTACCTGAATGACCACC<br>Reverse: AACCAGCGGTTGAAGCGTTCCT
25	BCL2	Forward: GGTGGGGTCATGTGTGTGG<br>Reverse: CGGTTCAGGTACTCAGTCATCC
26	CDKN1B	Forward: ATCACAAACCCCTAGAGGGCA<br>Reverse: GGGTCTGTAGTAGAACTCGGG
27	CDKN1A	Forward: CGATGGAACTTCGACTTTGTCA<br>Reverse: GCACAAGGGTACAAGACAGTG
28	CDK2	Forward: CCAGGAGTTACTTCTATGCCTGA<br>Reverse: TTCATCCAGGGGAGGTACAAC
29	CDK4	Forward: ATGGCTACCTCTCGATATGAGC<br>Reverse: CATTGGGGACTCTCACACTCT
30	CDK6	Forward: CCAGATGGCTCTAACCTCAGT<br>Reverse: AACTTCCACGAAAAAGAGGCTT
31	CCND1	Forward: GCTGCGAAGTGGAAACCATC<br>Reverse: CCTCCTTCTGCACACATTTGAA
32	CCND2	Forward: ACCTTCCGCAGTGCTCCTA<br>Reverse: CCCAGCCAAGAAACGGTCC
33	GAPDH	Forward: ACAACTTTGGTATCGTGGAAGG<br>Reverse: GCCATCACGCCACAGTTTC
34	STAT3 3'UTR Mutation	Forward: CACATGGGGGAAGCAGCGCACTCCGAGGTCAA<br>Reverse: TTGACCTCGGAGTGCGCTGCTTCCCCCATGTG
35	IL-6 3'UTR Mutation	Forward:TAAAAATATGTATAAGTTAGCCATTTATTTTAAGCAAGCCTACACTTTCCAAGAAATGATCTGGC<br>Reverse:GCCAGATCATTTCTTGGAAAGTGTAGGCTTGCTTAAAATAAATGGCTAACTTATACATATTTTTA
